# Cochlear implants: a transformative technology

**DOI:** 10.2471/BLT.19.020319

**Published:** 2019-03-01

## Abstract

Cochlear implants bring sound to people living with permanent hearing loss. But making them accessible to all in need is a major challenge. Andrey Shukshin reports.

Stanislav Rubinstein lost his hearing on New Year’s Eve, 2009. The then 20-year-old power company worker had been in an accident and had received treatment for severe burns and spinal damage. But it was not the accident that affected his hearing, it was the antibiotics he was given in hospital.

“It happened very quickly,” says Stanislav, who now lives just outside Moscow in the Russian Federation. “December 31, 2009 was the last day I heard anything with my ears.”

Five months and 18 operations later, including multiple skin grafts, Stanislav left the hospital able to walk, but cut off from the world of sounds, including the sound of his beloved guitar. He was also cut off from the profession he enjoyed. “I was told I would have to apply for a job as a garment maker,” he says.

Three years later he learned of a device known as a cochlear implant.

Cochlear implants are hearing devices comprised of an external microphone and speech processor worn just behind the ear that converts sound into electrical stimuli, which are captured electromagnetically by a surgically implanted antenna. The antenna directs the signal to the internal electrodes, which in turn stimulate the auditory nerve.

“They are truly ingenious devices,” says Dr Shelly Chadha, who heads the World Health Organization’s (WHO) work on prevention of deafness and hearing loss. “The direct stimulation of the auditory nerve allows the device to overcome hearing loss that occurs when there is damage to the cochlea. This is the most common type of permanent hearing loss and it cannot usually be medically or surgically corrected. So for people with this particular problem, cochlear implants are the only hope of hearing.”

Unfortunately, not everyone who could benefit from a cochlear implant gets one. As Chadha explains, there are several reasons for this, the most obvious being their cost. A cochlear implant, including the cost of implant surgery, can be as high as US$ 50 000 in high-income countries, while the external components (transmitter and speech processor) can cost around US$ 9000. 

No generic version of cochlear implants exists, and the four main manufacturers have kept their prices high even in low- and middle-income countries. In the Russian Federation, for example, a cochlear implant costs up to US$ 18 000, equivalent to roughly two years of the national average income.

“It is possible that costs will come down as new producers come into the market,” says Chadha, noting that domestic production in China supplies government programmes there while the federal government of India is discussing the possibility of manufacturing cochlear implants to support the National Programme for Prevention and Control of Deafness.

“Post-operative rehabilitation may take five to seven years and sometimes a lifetime.”

Victoria Mukhina

But even if the price of cochlear implants does come down, it is unlikely to have a major impact on the overall cost of implementing effective cochlear implant programmes and thus the availability of devices for people who need them. 

For example, the Russian government started supporting cochlear implantation with the implantation of two devices in 1987 increasing to 100 implantations in 2005. Despite a sharp economic downturn since 2008, the government has continued to support implantation, funding around 1000 cochlear implants a year out of the federal budget. In the absence of official statistics, it is difficult to establish how close this support comes to meeting the demand. However, according to Dr George Tavartkiladze, founder and director of the National Research Centre for Audiology and Hearing Rehabilitation, demand currently stands at around 3000-35 000 implants annually.

Roughly 90% of the implants are reserved for children. Maternity hospitals with a capacity of more than 1000 births per year, regional audiological centres and pediatric outpatient clinics are equipped for newborn hearing screening. 

Tavartkiladze estimates that initial screening coverage is close to universal (98%) in the majority of the federation’s 85 administrative regions (krai). He points out however, that coverage of second stage screening, which involves a full diagnostic test, is done only in the regional audiological centres, and currently stands at around 80%.

Why this emphasis on treating children? Tavartkiladze explains that children are considered more likely to benefit from the procedure than adults who have been deaf from birth. However, adults who have lost their hearing due to illnesses such as meningitis or to head injury, are eligible to have an implant fitted within a few months.

According to Tavartkiladze, almost all implantations are done at six federal institutions, three of which are located in Moscow, two in Saint Petersburg, and only one, the Federal Siberian Research and Clinical Center, in Krasnoyarsk. “The operation itself has become a routine procedure,” he says, underlining the high success rate achieved by surgeons and their teams.

While the Russian Federation’s cochlear implant programme is clearly laudable in many ways, it also has some important limitations. To begin with, in most cases, the government only funds one implant per person (only around 10% of patients receive two implants under the state programme, including all patients with meningitis-related deafness). This limits the recipient’s ability to participate in everyday life. “The benefit of using implants on both ears, so-called binaural implantation, is in gaining the ability to localize sound and in significantly higher speech intelligibility in acoustically difficult environments, such as noisy rooms,” says Victoria Mukhina, head of Tosha&Co, a private rehabilitation centre for children with cochlear implants, which is located in Fryazino just outside Moscow.

Mukhina believes it is vital that the government support binaural implantation. “It’s a question of resources,” she says. “We must get together and get the government to fund two implants on a massive scale.” She also believes that it is essential that the government expand implantations for adults, pointing out that in most developed countries adults account for more than half of the implantations undertaken.

The fact that the federal government tends to fund only the first post-operative rehabilitation session is also a matter of concern. The Moscow region benefits from the Federal Centre for Rehabilitation of Children with Hearing Loss, where all children with cochlear implants can get two weeks of rehabilitation sessions free of charge, but outside the Moscow region no such service exists.

This is a problem, as Mukhina explains. “The implant operation itself only addresses part of the patient’s needs,” she says, noting that when the implant is switched on, the external speech processor does not result in the sudden restoration of hearing. The sounds produced by the speech processor have to be reinterpreted.

“Cutting-edge technologies are great, but they need to be supported by a properly trained and motivated workforce.”

Alexander Ivanov

“This process is easier for the adults and children who lose their hearing after they have already mastered speech. For those who become deaf before they have learned how to speak or who are born with hearing loss, the earlier they get the implant, the better the results they can achieve.” Mukhina says. “Post-operative rehabilitation may take five to seven years and sometimes a lifetime,” she adds.

There are currently five main medical institutions undertaking medical rehabilitation, and once again, services are highly centralized, with four of the centres located in Moscow, and one in Saint Petersburg. 

Sufficient staff are also needed. Learning how to hear and speak can require the support of audio-therapists, psychologists and neurologists. “Cutting-edge technologies are great, but they need to be supported by a properly trained and motivated workforce,” says Alexander Ivanov, head of rehabilitation at the Russian Deaf Association. “To focus on just one speciality, we currently have a 30% shortage of audiologists across the country.”

 “I have a feeling that officials thought cochlear implants were this wonderful, quick-fix, high-impact solution,” says Ivanov, “but that is not how things work.”

Stanislav Rubinstein would agree, in part. It took him seven years to get back to full participation in everyday life; but he would consider “wonderful” and “high-impact” entirely appropriate descriptions of the devices he now proudly wears.

Today, apart from those devices there is no indication of his deafness. After receiving a job offer from the local distributor of a major international cochlear implant producer, he moved to a town outside Moscow, where he is now working as a technical assistant.

“I am so happy with my implants that if somebody told me there is a wonder surgeon who could try and get my natural sense of hearing back, I wouldn’t go for that. I lead a normal life, I communicate freely, I have a great job, I enjoy music just like before and I can play my beloved guitar like I used to,” he says.

**Figure:**
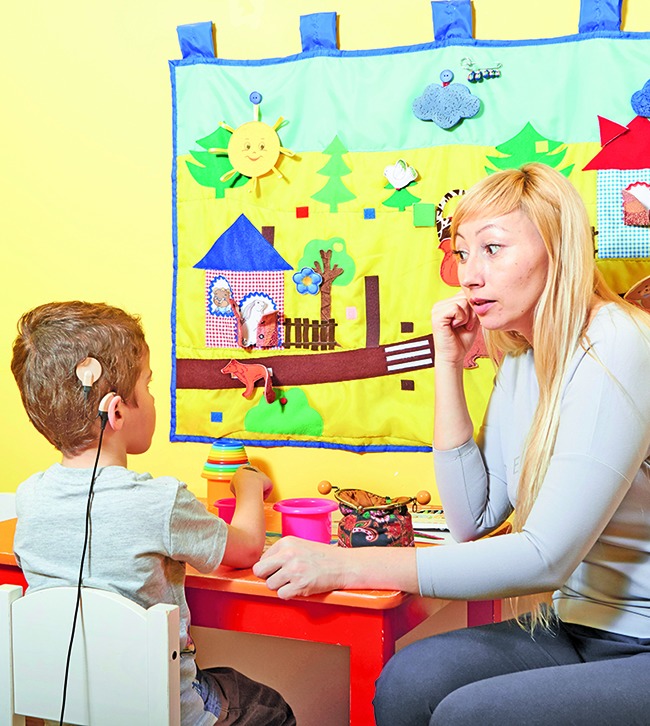
A speech therapist works with a young boy wearing a cochlear implant in a rehabilitation centre, Fryazino, Russian Federation.

Courtesy of Tosha & Co/Svetlana Kulekhina

**Figure:**
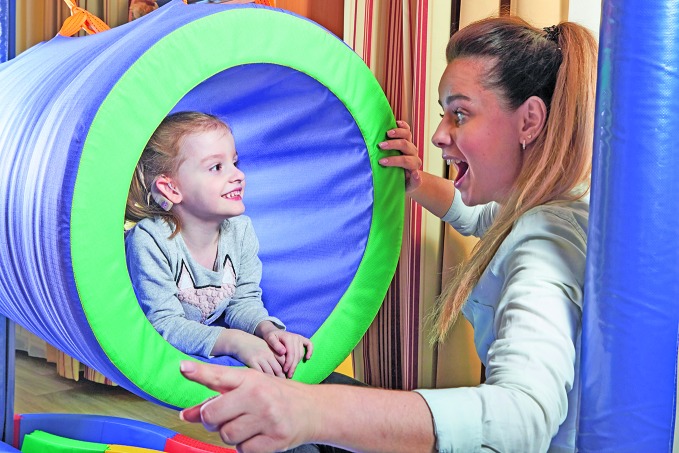
A speech therapist works with a young girl wearing a cochlear implant in a rehabilitation centre, Fryazino, Russian Federation.

Courtesy of Tosha & Co/Svetlana Kulekhina

